# Caretakers’ Perception towards Using Zinc to Treat Childhood Diarrhoea in Rural Western Kenya

**DOI:** 10.3329/jhpn.v31i3.16823

**Published:** 2013-09

**Authors:** George A. Otieno, Godfrey M. Bigogo, Bryan O. Nyawanda, Frances Aboud, Robert F. Breiman, Charles P. Larson, Daniel R. Feikin

**Affiliations:** ^1^Centre for Global Health Research, Kenya Medical Research Institute, Kisumu, Kenya; ^2^International Emerging Infections Program, Centers for Disease Control and Prevention, Nairobi, Kenya; ^3^McGill University, Montreal, Canada; ^4^University of British Columbia, Vancouver, Canada

**Keywords:** Acceptability, Community treatment, Diarrhoeal disease, Zinc, Kenya

## Abstract

Zinc treatment for diarrhoea can shorten the course and prevent future episodes among children worldwide. However, knowledge and acceptability of zinc among African mothers is unknown. We identified children aged 3 to 59 months, who had diarrhoea within the last three months and participated in a home-based zinc treatment study in rural Kenya. Caretakers of these children were enrolled in two groups; zinc-users and non-users. A structured questionnaire was administered to all caretakers, inquiring about knowledge and appropriate use of zinc. Questions on how much the caretakers were willing to pay for zinc were asked. Proportions were compared using Mantel-Haenszel test, and medians were compared using Wilcoxon Rank Sum test. Among 109 enrolled caretakers, 73 (67%) used zinc, and 36 (33%) did not. Sixty-four (88%) caretakers in zinc-user group reported satisfaction with zinc treatment. Caretakers in the zinc-user group more often correctly identified appropriate zinc treatment (98%-100%) than did those in the non-user group (64−72%, p<0.001). Caretakers in the zinc-user group answered more questions about zinc correctly or favourably (median 10 of 11) compared to those in the non-user group (median 6.3 of 11, p<0.001). Caretakers in the zinc-user group were willing to pay more for a course of zinc in the future than those in the non-user group (median US$ 0.26, p<0.001). Caretakers of children given zinc recently had favourable impressions on the therapy and were willing to pay for it in the future. Active promotion of zinc treatment in clinics and communities in Africa could lead to greater knowledge, acceptance, and demand for zinc.

## INTRODUCTION

Approximately half of the estimated 7.7 million deaths in children aged less than five years in 2010 occurred in sub-Saharan Africa. About 19% of childhood deaths in Africa occur due to diarrhoea (1-3). Some of the risk factors of diarrhoeal deaths are ingestion of unsafe water, lack of access to proper sanitation, and deficiency in zinc and vitamin A (2-6). Both preventive and therapeutic approaches to diarrhoea have the potential to prevent diarrhoea-related deaths (7). One strategy that has been shown to both treat and prevent diarrhoea is zinc supplementation (8-14). Zinc treatment during diarrhoea episodes has been shown to decrease duration and severity, and to prevent subsequent diarrhoea and acute lower respiratory infections over the ensuing 2-3 months (8,10). Using zinc to treat diarrhoea has the potential to avert up to an estimated 400,000 deaths annually and has been recommended for treatment of children with diarrhoea in developing countries by UNICEF and WHO (11). Research carried out in Bangladesh, India, and Mali has shown that zinc is well-accepted, and most caretakers are willing to buy zinc (4,14-17). While zinc has increasingly been used in diarrhoea treatment in Asian countries for several years, it has not widely been instituted in Africa (14).

A policy to use zinc to treat children with diarrhoea was adopted by the Ministry of Health of Kenya in 2008. In much of Kenya, healthcare utilization for common illnesses is poor. The 2009 Demographic and Health Survey for Kenya found that only 49% of diarrhoeal episodes in Kenyan children resulted in a visit to a health facility or care provider (18). As such, the majority of diarrhoeal episodes in Kenyan children are not treated with zinc. To inform the approaches addressing this problem, we carried out a cluster-randomized study of the uptake and impact of using zinc to treat diarrhoeal children aged less than five years in their homes in rural western Kenya. The study was conducted from December 2007 to April 2009. Within this study population, we designed a nested substudy to determine if there were any differences between caretakers (usually mothers) who used zinc for their children's last diarrhoeal episode and those who did not, in terms of their acceptance, knowledge, and willingness to pay for zinc. Understanding how caretakers accept zinc will inform decision-makers in the ministries of health in African countries about optimal ways to introduce zinc into their healthcare systems.

## MATERIALS AND METHODS

### Study setting

Since 2006, International Emerging Infections Program of the Centers for Disease Control and Prevention (CDC), in collaboration with Kenya Medical Research Institute (KEMRI), had a population-based infectious disease surveillance (PBIDS) in Rarieda division in rural western Kenya (19). The area is one of the poorest in Kenya where most people practise subsistence farming and where rates of malaria, HIV, and childhood infections are high (19,20). PBIDS includes 33 villages, with an approximate enrolled population of 25,000, among whom 3,100 were children aged <5 years. The enrolled households were visited every two weeks by fieldworkers who inquire about key symptoms of major infectious diseases, such as diarrhoea, respiratory infections, and malaria. All PBIDS participants were entitled to free high-quality healthcare at a centrally-located mission hospital called Lwak Hospital.

### Study of zinc treatment at home

Within 33 villages under the PBIDS area, we undertook a cluster-randomized controlled trial of zinc-use in diarrhoea treatment at home for children. Villages were stratified by size and randomly selected within the strata. All caretakers of children from 16 villages received a blister pack of 10 zinc tablets and 3 sachets of low-osmolarity ORS every 2 months at home. Caretakers of children from 17 villages received only 3 sachets of low-osmolarity ORS every 2 months. Caretakers in all villages were counselled on the indications and administration of zinc and ORS by trained community-based staff called ‘village reporters’. Village reporters are often traditional birth attendants but are not necessarily community health workers. Village reporters underwent a week-long training at the beginning of the study on zinc-use and received a two-day refresher training halfway through the study. Caretakers of children in both the groups were advised to take their children to Lwak Hospital when their children were ill. At Lwak Hospital, children with diarrhoea received zinc if not already taken it at home, regardless of whether they were assigned to the home zinc treatment group or the non-home zinc treatment group. The home zinc treatment study began in December 2007 with the last zinc distribution in August 2009 at home. At the start of the study, zinc had not become available in public clinics, although the Ministry of Health had decided to adopt zinc for treatment of diarrhoea. The general population had little knowledge of or experience with zinc. By the end of the study, zinc had become available in most clinics under the Ministry of Health but not in shops or informal pharmacies.

### Zinc acceptability study

This study was carried out between November 2009 and January 2010 among caregivers of children aged 3-59 months enrolled in the main home zinc treatment study described above. The caretakers were identified through routine biweekly visits for the PBIDS. A list of all children who experienced diarrhoea in the last three months was generated from PBIDS data. In total, 347 children were categorized into two groups: those whose last diarrhoeal episode was treated with zinc (zinc-users) and those whose last episode was not treated with zinc (non-users of zinc). To minimize recall bias, we selected 109 caretakers of those children who had the most recent diarrhoeal episode. Non-users of zinc only referred to diarrhoeal episodes in the past 3 months, and it is possible that those children were treated with zinc for previous diarrhoeal episodes.

### Sample-size

As this was a substudy of the randomized trial, we had only a limited budget to interview approximately 100 caretakers of the study children. We determined what difference we could detect, given the power of enrolling this number of caretakers. We found that we could detect a doubling of the willingness to pay from 30% to 60%, assuming a p value of 0.05, power of 80%, and a ratio of 2 zinc-users for each non-user. We felt that 30% was a reasonable assumption for willingness to pay in the non-zinc group and also felt that a doubling of the willingness to pay constituted a reasonable increase that had significant public-health impact. We oversampled zinc-users since one of the main objectives was to assess the experience of zinc-use among caretakers. Since we wanted to know if any zinc-use, rather than assignment to the home zinc treatment group, influenced willingness to pay, we enrolled approximately half of the participants, in both zinc-user and non-user groups, from the villages under home zinc treatment and half from the villages under non-home zinc treatment (Table 1). The final sample included 73 zinc-users (37 home zinc treatment and 36 non-home zinc treatment groups) and 36 non-users (18 home zinc treatment and 18 non-home zinc treatment groups).

### Data collection

Data were collected using a structured questionnaire. We asked a series of questions about knowledge and acceptability of zinc. Among these, we asked 11 Likert-scale questions, including responses ranging from ‘agree’, ‘partially agree’, ‘disagree’ to ‘don't know’. Questions could be framed positively (e.g. zinc stops diarrhoea quickly or soon) or negatively (e.g zinc worsens the severity of diarrhoea). Participants were asked to agree or disagree with positively- and negatively-framed questions. Questions on willingness to pay, treatment, and healthcare-seeking for diarrhoea were also asked. Interviewers were secondary school graduates who underwent a week-long study-specific training on questionnaire administration. Interviews were conducted in the local language Dholuo, using agreed upon standardized phrasing of questions. Data were collected on scannable forms (Teleforms, Cardiff Inc.). Scanned data were reviewed by specialists and corrected where appropriate, or sent back to the field team for clarification.

**Table 1.** Comparison of demographic characteristics of zinc-users and non-users, Rarieda, Kenya, 2009

Parameter	Zinc-users (N=73)	Non-users (N=36)	p value
Home zinc treatment group, N (%)	37 (50.7)	18 (50.0)	0.95
Age of children in months (median)	21.6	21.6	0.99
Male children, n (%)	44 (60.3)	19 (52.8)	0.46
Age of mother in years (median)	27.6	27.5	0.37
Mother finished primary school, n (%)	45 (61.6)	23 (65.7*)	0.68
Father has salaried employment, n (%)	7(9.6)	2 (5.6)	0.47
Lowest SES quintile† n (%)	9 (12.3)	4 (11.1)	0.85
2nd-lowest SES quintiles† n (%)	21 (28.7)	5 (13.9)	0.14

*One participant missing mother's education; †The SES score was derived using multiple component analysis (MCA) for all households in the surveillance area. The MCA was generated based on household characteristics and assets, namely occupation of household head, primary source of drinking-water, main source of cooking-fuel, in-house possession (lantern lamp, sofa, radio, bicycle, and TV), and livestock ownership (goats, cattle, donkeys, pigs, and sheep)

### Statistical analysis

Data were analyzed using SAS (version 9.2, Cary NC) and Epi Info (version 2002). Proportions were compared using the Mantel-Haenszel test. During analysis of Likert-scale questions about zinc, responses done correctly or favourably towards zinc in terms of acceptability were given a score of 1, ‘partially agree’ responses were given a score of ½, and incorrect responses or ‘don't know’ were given a score of 0. Due to non-normal distribution of responses, median scores between zinc-users and non-users were compared using the Wilcoxon Rank Sum test.

### Ethical approval

The protocol was approved by the ethical review boards of KEMRI and CDC. Each participant signed a written informed consent form.

## RESULTS

Of the 3,430 children enrolled in the main home zinc treatment study, 347 were reported from PBIDS to have had diarrhoea in the last three months, out of which we enrolled a sample of 109 (31%) caretakers of children with the most recent diarrhoeal episodes. By design, approximately half (n=55) were enrolled from the villages that received zinc at home and half (n=54) from villages that were not provided zinc at home. The median time from the most recent diarrhoeal episode to the time of enrollment was 63 days for zinc-users and 60 days for non-users (p=0.09). Demographic characteristics were similar between zinc-users and non-users (Table 1).

### Treatment with zinc

Among the 109 enrolled caretakers, 73 reported using zinc and 36 did not use zinc for their children's most recent diarrhoeal episode. Among the 73 zinc-users, 18 (25%) received zinc at home, 48 (66%) obtained zinc at a clinic, 5 (6%) from both clinic and at home, and 2 (3%) from other sources (e.g. neighbours). Other treatments used for diarrhoea were ORS, antibiotics, herbal medicines, and intravenous fluids (Table 2). Zinc-users had significantly lower use of antibiotics than non-users of zinc (20% among zinc-users vs 69% among non-users of zinc, p<0.001).

**Table 2.** Care-seeking and treatments administered besides zinc for the most recent episode of diarrhoea, Rarieda, Kenya, 2009

Treatment other than zinc	Zinc-users (N=73) n (%)	Non-users (N=36) n (%)	p value
ORS	63 (87)	27 (75)	0.09
Antibiotics	15 (20)	25 (69)	<0.001
Intravenous fluids	14 (19)	5 (13)	0.49
Prayer	2 (2.8)	3 (8.3)	0.20
Herbal medicine	13 (18)	8 (22)	0.60
Visited clinic	53 (73)	18 (50)	0.02

Among the caretakers of zinc-users, 61 (84%) reported taking the full 10-day course. The most common reasons for stopping zinc before taking all the tablets were that their children were better (n=8) or they thought they were supposed to take fewer than all 10 tablets (n=4); none indicated that they stopped because of unpleasant taste or side-effects. Among the zinc-users, 71 (97%) reported to have improved appetite after initiating zinc, with most (86%) within the first 4 days of treatment. All 73 (100%) caretakers of zinc-users reported that the diarrhoea stopped within 10 days, and 80% reported that diarrhoea stopped within the first 4 days of starting zinc. Sixty-two caretakers (86%) of zinc-users reported that they dissolved the zinc tablet in breastmilk or water before giving it to the child, and 61 (84%) reported that it was not difficult to administer zinc. The main difficulty reported was that the child “did not want to take the zinc tablet”, although the specific reasons why they did not want were not further clarified in the questionnaire.

Twenty-one (29%) zinc-users vomited after taking the zinc tablet, of whom 17 (81%) vomited only after the first dose.

Thirty-six caretakers did not use zinc when their children had diarrhoea, reporting the following reasons: 16 (44%) did not know where to obtain zinc from (5 in home zinc villages and 11 in non-home zinc villages), 15 (42%) were not prescribed zinc from clinic, 4 (11%) were unsure about how to administer zinc, and 1 (2.8%) did not know about the existence of zinc treatment.

### Satisfaction with zinc

Among the caretakers who gave zinc to their children, 50 (69%) reported that they thought the zinc tastes better than other medicines, 22 (30%) felt the taste was similar to other medicines, and one (1.4%) thought the taste was worse than other medicines. Sixty-four caretakers (88%) reported being satisfied with the zinc treatment.

### Knowledge of zinc

When caretakers of zinc-users were asked for which conditions they would recommend zinc for their children, all 73 (100%) caretakers of zinc-users correctly stated that zinc should be used for watery diarrhoea compared to 26 (72%) of non-users (p<0.001, Table 3).

Among the caretakers who used zinc, 71 (97%) correctly reported that zinc should be used for bloody or mucousy diarrhoea compared to 23 (64%) of the non-users of zinc (p<0.001). The differences between zinc-users and non-users were found among both those in the home zinc and the non-home zinc groups (Table 3). There was no difference in the percentage of caretakers who said they would (inappropriately) use zinc for teething—13 (18%) of zinc-users vs 7 (19%) of non-users (p=0.8). Two zinc-users would consider use of zinc inappropriate for fever compared to one non-user (p=0.99). No caretakers used zinc for cough.

**Table 3.** Caretakers’ knowledge about appropriate zinc-use based on whether they had used zinc and whether they were assigned to home zinc treatment villages, Rarieda, Kenya, 2009

Reason for zinc-use	Overall			Home zinc villages			Not home zinc villages		
	Zinc used	No zinc used	p value	Zinc used	No zinc used	p value	Zinc used	No zinc used	p value
	(n=73)	(n=36)		(n=37)	(n=18)		(n=36)	(n=18)	
Zinc should be used for:									
Watery diarrhoea	73 (100)	26 (72)	<0.001	37 (100)	12 (67)	<0.001	36 (100.0)	14 (78)	0.003
Mucous/bloody diarrhoea	71 (97)	23 (64)	<0.001	37 (100)	11 (61)	<0.001	34 (94)	12 (67)	0.007
Fever	2 (2.7)	1 (2.8)	0.99	2 (5.4)	1 (5.6)	0.99	0	0	-
Cough	0	0	-	0	0	-	0	0	-
Teething	13 (18)	7 (19)	0.84	6 (16)	3 (17)	0.99	7 (19)	4 (22)	0.99

### Acceptability of zinc

For the 11 Likert-scale questions on acceptability of zinc, a score of 11 meant that the caretaker had the most favourable impression on zinc, and a score of 0 indicated the most unfavourable impression (Table 4). Caretakers of zinc-users had a median score of 10 while those of non-users had a median score of 6.3 (p<0.001). There were no significant differences in the scores between caretakers from home zinc and non-home zinc villages.

### Willingness to pay

Caretakers of all 73 (100%) zinc-users said they would be willing to pay for zinc in the future; the median price they would be willing to pay for a 10-day course of therapy was 20 Kenya Shillings (US$ 0.26), ranging from 5 KSh (US$ 0.06) to 200 KSh (US$ 2.60). Also, all 36 (100%) caretakers of non- users of zinc reported a willingness to pay for zinc; the median price was 20 Kenya Shillings (US$ 0.26), ranging from 10 KSh (US$ 0.13) to 40 KSh (US$ 0.52) (Figure). Comparison of the distributions around the median amount among groups revealed that caretakers of zinc-users were willing to pay more (p<0.001); 101 (93%) respondents preferred zinc to be available in shops or through community health workers rather than only in clinics.

## DISCUSSION

Our study demonstrated that caretakers of young children with diarrhoea in rural western Kenya, who were treated with zinc, had a favourable impression on zinc treatment for diarrhoea. Most were ‘satisfied’ with the use of zinc in their children and found zinc easy to use. This general positive perception of zinc among mothers and other caregivers was similar to what has been observed in Bangladesh and Mali (5,6,16,21-23). The most negative impression on zinc was that it caused vomiting, although this was limited to the first dose in most cases—a known adverse event with zinc treatment (24) and did not result in stopping the treatment course for any patient in our study.

Knowledge about appropriate zinc-use differed significantly between zinc-users and non-users. Moreover, zinc-users scored higher than non-users on a set of questions that evaluated knowledge and acceptability of zinc. In contrast, we saw no difference in the percentage of zinc-users and non-users who correctly identified inappropriate use of zinc (e.g. for fever and cough), which was high in both the groups. Of note, however, up to 20% of caretakers thought that zinc was an appropriate treatment for diarrhoea during teething, a notion that might derive from the local belief that teething children develop diarrhoea, a belief also held in rural Mali (4-6). Importantly, it was rare for parents to respond that zinc would be useful to treat fever, perhaps allaying fears in this malaria-endemic area that parents might delay seeking appropriate treatment for malaria by using zinc instead (4-6). Critically, zinc-users were less likely to have received antimicrobial therapy. Given that the vast majority of childhood diarrhoeal episodes within the study area were ‘watery diarrhoea’, antimicrobial therapy would be unnecessary. Thus, availability of zinc therapy may be useful in reducing inappropriate use of antimicrobial drugs. Despite this finding, our study was not designed to specifically address decisions about antimicrobial-use, and further formative work is needed to clarify this.

**Table 4.** Evaluation of acceptability and knowledge of zinc-use to treat diarrhoea among zinc-users and non-users by number of questions answered correctly or positively, defined as correctly agreeing or disagreeing with the question as phrased, Rarieda, Kenya, 2009

Statement in the questionnaire	Zinc-users (N=73) n (%)	Non-users (N=36) n (%)	p value
1. Zinc stops diarrhoea quickly or soon	73 (100.0)	16 (44.4)	<0.001
2. Zinc worsens the severity of diarrhoea	71 (97.3)	19 (52.8)	<0.001
3. Zinc protects the child from getting another episode soon	48 (65.8)	11 (30.6)	0.001
4. Zinc is difficult to administer	69 (94.5)	23 (63.9)	<0.001
5. Zinc improves the appetite of the child	67 (91.8)	13 (36.1)	<0.001
6. Zinc is not good as alternative home remedies for diarrhoea	71 (97.3)	26 (72.2)	<0.001
7. Zinc is not necessary if diarrhoea is mild	61 (83.6)	14 (38.9)	<0.001
8. I would not use new drugs such as zinc if recommended by my care provider	71 (97.3)	32 (88.9)	0.07
9. Zinc has no side-effect	70 (95.9)	20 (55.6)	<0.001
10. Zinc is known and used by mothers in my village	58 (79.5)	24 (66.7)	0.15
11. Zinc is highly recommended by healthcare providers	67 (91.8)	26 (72.2)	0.01
Overall score – Median*	10.0	6.3	<0.001

*1 point for correct answer, ½ point for ‘partially agree’ and 0 for incorrect answer or ‘don't know’

**Figure.**
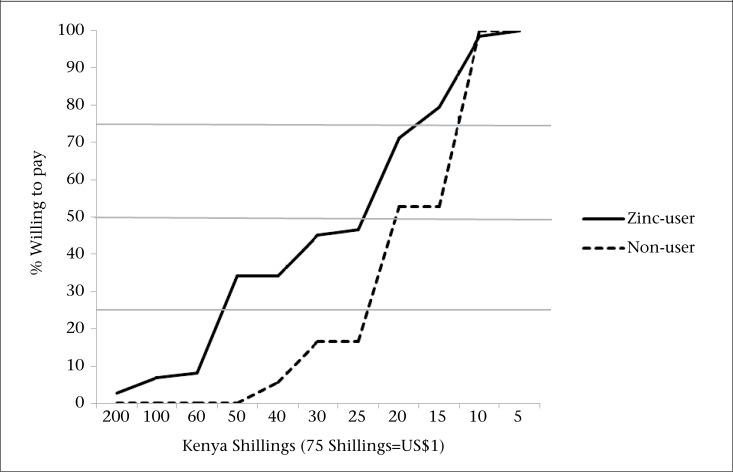
Cumulative percentage of willingness to pay for zinc treatment among caretakers of children who used zinc and children who did not for recent episodes of diarrhoea, Rarieda, Kenya, 2009

There could be several reasons for the greater level of knowledge among caretakers of zinc-users than non-users. First, it is possible that those who knew more about the benefits and indications for zinc, either from education at the home as part of the home zinc treatment study or from education at the clinic where they were given zinc, were more likely to be zinc-users because of that knowledge. This is the desired outcome of an educational programme. Second, according to the selection criteria, zinc-users used zinc to treat a recent episode of diarrhoea. The majority of them obtained zinc from the health clinic, and so had recent contact with a healthcare worker who likely explained to them the indications for zinc-use. If this is the case, it suggests that education given in the health facilities about zinc is retained, perhaps as long as 60 days after the encounter. Third, the association between knowledge and use of zinc might be confounded by another variable. While the characteristics of zinc-users and non-users in terms of maternal age and education, father's employment, and socioeconomic status were similar between the two groups, it is possible that there were other differences between the zinc-users and non-users that we could not identify. For example, mothers who are more aware of health conditions seek treatment for their sick children and also know more about those treatments that they seek, such as zinc.

There was a high level of overall general knowledge about zinc-use in this study population in both the study areas (including areas where zinc was not provided to the caretakers by village reporters). All caretakers had a visit from a village reporter every two months to deliver ORS and some education on diarrhoea. All study participants could obtain zinc and information about zinc from Lwak Hospital and clinics under the Ministry of Health. Another possibility is that knowledge about zinc was prevalent in the community through most clinics under the Ministry. Therefore, many caretakers might have already been exposed to zinc treatment in the clinics.

All participants, both caretakers of zinc-users and non-users, said they would be willing to pay for zinc. The median amount (approximately US$ 0.26) they said they would be willing to pay for a 10-day course of therapy, was comparable to the cost of zinc in a community-based zinc introduction strategy in Mali (US$ 0.19) and in Bangladesh's scale-up campaign (US$ 0.25) (16,22,23). Zinc-users were willing to pay more than non-users.

### Limitations

Besides possible confounding, our study had several other potential limitations. First, the study population was not representative of the greater population, who would not have had the bimonthly mini-educational sessions that were given at home as part of the randomized controlled trial. Second, there might have been selection bias in that caretakers of children with recent diarrhoeal episodes, who were enrolled in this study, might have differed in their knowledge and attitudes about zinc from caretakers of children without recent diarrhoeal episodes. Third, we labelled persons as non-users of zinc if they did not use zinc for their last episode, although they might have used it in prior episodes. Therefore, caretakers of children in the non-user group might have had past experience with zinc-use. If this were the case, however, this should have biased the findings towards the null rather than lead to the differences between groups that we found.

### Conclusions

Knowledge and acceptability of zinc was high among caretakers who gave their children zinc for a recent diarrhoeal episode. Although what caretakers said and did might differ, this favourable impression on zinc, along with a stated willingness to pay for the treatment, suggests that availability of zinc in shops or in the community through community health workers would likely result in an increase in zinc-use for treatment of diarrhoea in rural western Kenya. In this part of Africa, the majority of diarrhoeal episodes in children are treated outside the formal healthcare system, which is currently the only place to obtain zinc from (25). Where healthcare utilization remains low, having access to zinc and education about diarrhoea management outside the formal health sector could increase the use of this important curative and preventative treatment.

## ACKNOWLEDGEMENTS

This work was supported by funds from Bill & Melinda Gates Foundation through a collaboration with icddr,b. We would like to acknowledge the Kenya Medical Research Institute for making this study possible and permission given by the Director to publish this work. We thank the people of Rarieda for their support and participation in this project. We would like to extend particular thanks for contributions to this study by the entire field-level data-collectors, supervisors in Rarieda, and the Ministry of Health. We also thank Dr. Deron Burton for his support and advice.
